# Individual-Level Fitness and Absenteeism in New York City Middle School Youths, 2006–2013

**DOI:** 10.5888/pcd15.170152

**Published:** 2018-01-11

**Authors:** Emily M. D’Agostino, Sophia E. Day, Kevin J. Konty, Michael Larkin, Subir Saha, Katarzyna Wyka

**Affiliations:** 1 City University of New York Graduate School of Public Health and Health Policy, New York, New York; 2 New York City Department of Health and Mental Hygiene, Office of School Health, New York, New York; 3 New York City Department of Education, Office of School Health, New York, New York

## Abstract

**Introduction:**

Youth health-related fitness positively affects academic outcomes, although limited research has focused on the relationship between fitness and school absenteeism. We examined the longitudinal association between individual children’s fitness and lagged school absenteeism over 4 years in urban middle schools.

**Methods:**

Six cohorts of New York City public school students were followed from grades 5 through 8 (school years 2006–2007 through 2012–2013; n = 349,381). A 3-level longitudinal generalized linear mixed model was used to test the association of change in fitness composite percentile scores and 1-year lagged child-specific days absent.

**Results:**

Adjusted 3-level negative binomial models showed that students with a more than 20% increase, 10% to 20% increase, less than 10% increase or decrease, and 10% to 20% decrease in fitness from the year prior had 11.9% (95% confidence interval [CI], 7.2–16.8), 6.1% (95% CI, 1.0–11.4), 2.6% (95% CI, −1.1 to 6.5), and 0.4% (95% CI, −4.3 to 5.4) lower absenteeism compared with students with a more than 20% fitness decrease.

**Conclusion:**

Cumulative effects of fitness improvement could have a significant impact on child absenteeism over time, particularly in high-need subgroups. Future research should examine the potential for school-based fitness interventions to reduce absenteeism rates, particularly for youths who have fitness drop-offs in adolescence.

## Introduction

Youth physical activity and health-related fitness (henceforth fitness) positively affects academic outcomes (1,2), potentially acting through pathways involving enhanced cognition and memory (3) or improvements in both physical and psychosocial wellness (4,5). Fitness and physical activity are strongly associated, and frequent vigorous physical activities are likely to improve fitness (6). For example, daily physical activity of at least moderate intensity is associated with reduced clustering of cardiovascular risk factors in youths, including high blood pressure, insulin level, lipids, and adiposity (7). However, accelerometry data show that only 42% of children aged 6 to 11 years meet international physical activity recommendations for at least 60 minutes per day of moderate to vigorous physical activity (8). Although these rates are similar to rates in European countries (9), declines in physical activity are steeper from childhood to adolescence in the United States compared with declines in other nations (10). This national trend is also evident in New York City (NYC), where 40% and 20% of youths aged 6 to 12 and 14 to 18, respectively, meet physical activity recommendations (11,12).

Another established predictor of academic performance is school absenteeism (1,13), which may mediate the observed fitness–academic achievement association. Maintaining regular attendance, defined as missing fewer than 6 excused or unexcused days per year, predicts academic success (14). School absenteeism, regardless of reason, predicts poor academic achievement and is associated with poor school adjustment; alcohol, tobacco, and substance use; increased rates of teen pregnancy; juvenile delinquency; and both family and home–school disengagement (4,15,16). Fitness improvements may both directly and indirectly reduce absenteeism, working potentially through pathways involving self-esteem, physical health, mental health, and cognitive processing (3,4).

Limited research has examined the fitness–absenteeism relationship (4,5,17), demonstrating consistent inverse associations between fitness and school absenteeism. For example, Blom et al demonstrated that students with greater fitness had lower odds of more than 8 absences per year (odds ratio [OR], 3.31; 95% confidence interval [CI], 1.51–7.28 for students with 6 compared with less than 5 healthy fitness zones achieved) (5). Two other articles found significant crude associations between student physical activity and absenteeism (4,17). These studies drew predominantly from cross-sectional data and did not account for a range of potential confounders, including contextual factors that contribute to absenteeism and fitness. For example, neighborhood poverty contributes to parent–school engagement and youth fitness (18,19). Similarly, school size affects programs and policy toward school attendance and physical activity (20,21). The bulk of research on fitness and absenteeism is unable to support causal hypotheses given that temporality of exposure and outcome are not known. Nuanced research in this area that draws from individual-level measures collected over multiple years and includes school-level factors is necessary to better inform policy in support of increased school-based fitness programs.

We analyzed the longitudinal association between change in fitness and 1-year lagged absenteeism in 6 cohorts of NYC public school students based on year of initiating middle school and followed consecutively over 4 years (fitness change from grades 5 to 6, 6 to 7, and 7 to 8 paired with days absent per year for grades 6, 7, and 8, respectively) during a 7-year study period (2006–2007 through 2012–2013). We hypothesized that improvements in fitness (cardiorespiratory, muscular endurance, and muscular strength fitness composite percentile scores) would predict lower subsequent absenteeism.

## Methods

### Study population

Data were drawn from the NYC FITNESSGRAM (Fitnessgram) data set jointly managed by the NYC Department of Education (DOE) and Department of Health and Mental Hygiene (DOHMH) (22). It comprises annual fitness assessments collected by DOE for approximately 870,000 NYC public school students per year (grades K–12) starting in 2006–2007. This study was approved by the City University of New York and DOHMH institutional review boards.

**Figure 1:**
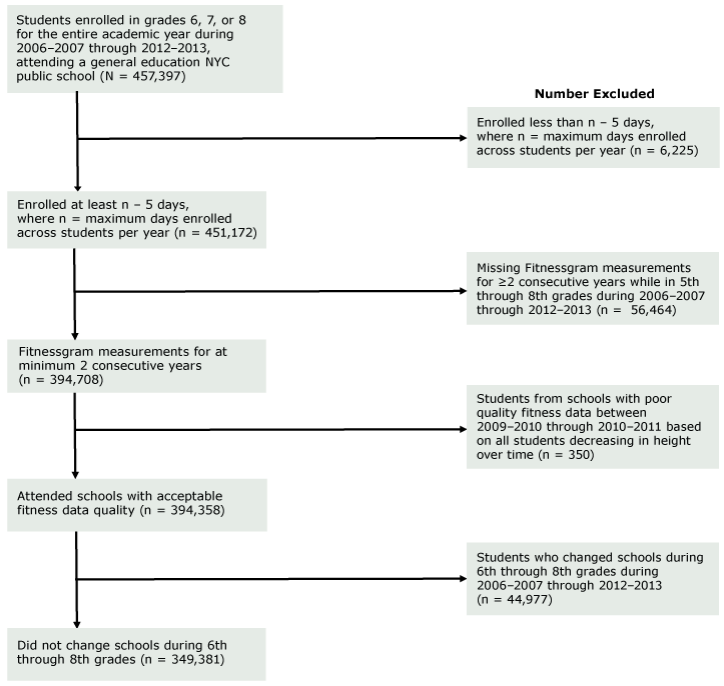
Sample selection flowchart for the association of fitness and absenteeism in New York City (NYC) public middle school students, 2006–2007 through 2012–2013.

The Fitnessgram is based on the Cooper Institute’s Fitnessgram, which has both strong reliability and validity (23). Fitnessgram performance tests provide a health assessment related to present and future health outcomes. NYC schools are mandated to have 85% or more of eligible students complete the test each year. Inclusion criteria for this study included enrollment in a NYC public school that collected Fitnessgram measurements for 2 or more consecutive years while in grades 6 through 8 during the study period (2006–2007 through 2012–2013) (see Figure 1 for sample selection flowchart). Student cohorts were defined based on year of initiating grade 6. Students were excluded (n = 6,225) if they were enrolled for less than n − 5 days per school year (where n is the maximum number of days enrolled across all students in each given year [n range: 292–297 days]) to ensure a consistent period of observation across school years with different total instructional days per year. Next, students were excluded if they did not take the Fitnessgram test for 2 or more consecutive years (n = 56,464), attended schools with poor-quality fitness data (n = 350), or changed schools during 6th through 8th grade (to be able to account for school clustering in the analysis; n = 44,977). After the above exclusions, the final sample of 6th through 8th graders included 349,381 unique students (51% male, 83% born in the United States, 38% Hispanic, 28% non-Hispanic black, and 16% non-Hispanic white; mean [standard deviation (SD)] school population = 541 [632]). Students in 6th, 7th, and 8th grades contributed 177,281, 220,769, and 186,135 student-years, respectively, across 624 schools.

### Measures

The primary exposure was a categorical variable representing age- and sex-specific percentage change in fitness composite percentile scores based on the sum of percentile scores for the Progressive Aerobic Cardiovascular Endurance Run (PACER), muscle strength and endurance (curl-up and push-up) tests (23). Scores were converted to percentiles to account for expected improvements in performance with increasing age and by sex. The fitness variable was categorized as more than 20% decrease, 10% to 20% decrease, less than 10% change, 10% to 20% increase, and greater than 20% increase in performance from the year prior, consistent with longitudinal research on fitness and academic outcomes drawing from the Fitnessgram data set (24).

The primary outcome variable for this analysis was student-level number of days absent per year. Annual enrollment and attendance records were matched to Fitnessgram results by a unique student identifier.

Adjusted models included sex, age, race/ethnicity, place of birth, socioeconomic status (SES), and school size. These covariates predict both fitness and absenteeism (4,20,21,24). Age at the time of height and weight measurement was treated as a continuous variable. Race/ethnicity was based on school enrollment forms completed by parents and grouped into 5 categories: Hispanic, non-Hispanic black, non-Hispanic white, Asian/Pacific Islander, and other. Place of birth (United States vs foreign country) was included as a covariate based on literature demonstrating that immigration status is predictive of physical activity (25) and school attendance (26). SES was defined as the percentage of households in the students’ school zip code living below the federal poverty threshold (low [<10%], medium [10%–20%], high [>20%–30%], and very high [>30%] poverty area) according to American Community Survey 2007–2012 data (27). School size classified schools, as per the literature, as small (<400 students) or nonsmall (≥400 students) (20).

Change in obesity status from the year prior (obese to not obese, consistently not obese, consistently obese, not obese to obese) was also included as a potential confounder based on the literature (4). Body mass index (BMI) is collected annually as a part of the Fitnessgram curriculum. Obesity was defined as having a BMI in the 95th percentile or higher for the same sex and age group using 2000 Centers for Disease Control and Prevention guidelines (28). Change in obesity status category was used in lieu of changes in BMI percentile to capture meaningful shifts in body composition associated with school outcomes (29).

### Statistical analysis

Descriptive statistics were computed to summarize sample characteristics. Next, trends in absenteeism (days absent) by fitness, grade, and demographics were examined.

Because observations were nested within students, nested within schools, mixed-model methods were used. Specifically, a series of crude and adjusted 3-level longitudinal generalized linear mixed models with random intercepts for student and school effects were fit to assess the fitness–absenteeism association while accounting for clustering and individual- and school-level confounders.

First, to determine the extent of variation in absenteeism at the school level, an unconditional model with random intercepts was fit to the data (model 1). The school-level intraclass correlation (ICC) was calculated as the ratio of the variance for the school divided by the sum of the 3 variance parameter estimates, represented as σ^2^_school_ / (σ^2^_student_ + σ^2^_school_ + σ^2^_ε_). Although univariate distributions for days absent demonstrated a long right-tailed Poisson distribution, the ICC was calculated based on a linear model given that the ICC definition is not well defined for Poisson models (30).

Next, the longitudinal association of change in fitness and lagged number of days absent per year was assessed by using a 3-level crude longitudinal negative binomial mixed model with random intercepts and the exposure, child-specific change in fitness from the year prior, as well as an offset term representing total instructional days per school year included in the model (model 2). Negative binomial models were used because data were overdispersed. β Coefficients represented the effects of the exposure, change in fitness on outcome, 1-year lagged number of days absent per year. Absenteeism rates were computed by calculating the incidence rate ratio, represented as exp(β).

Finally, potential individual- and group-level confounders were added to the model (model 3). Confounding variables included level-1 time-varying covariates for grade, year (to control for potential cohort effects), and change in obesity status from the year prior, level-2 covariates for individual sociodemographic factors (sex, race/ethnicity, place of birth), level-3 covariates for school size and SES, and interactions (grade*race/ethnicity, grade*sex, grade*place of birth, and SES*race/ethnicity).

In these analyses, students contributed fitness-change data for 5th to 6th, 6th to 7th, and/or 7th to 8th grades (n = 349,381 unique students; 675,318 observations). A 2-sided *P* value of less than .05 was considered significant. Analyses were performed using SAS version 9.4 software (SAS Institute, Inc).

## Results

Just under 40% of students had less than 10% change in fitness from the year prior, followed by greater than 20% increase (20%), greater than 20% decrease (19%), 10% to 20% increase (12%), and 10% to 20% decrease (12%) (Table 1). The mean (SD) number of days absent per year were highest among boys (11.0 [11.7]) and Hispanic (12.6 [12.9]) and non-Hispanic black (12.3 [13.1]) racial/ethnic groups (Table 2). Mean days absent were also highest among students who were born in the United States (11.3 [12.1]) compared with those who were born in a foreign country (11.1 [13.8]).

**Table 1:** Demographic and Fitness-Change Characteristics of New York City Public Middle School Students (N = 349,381), 2006–2007 Through 2012–2013

Characteristic	n^a^^,^ b (%)
**Sex**	
Male	177,355 (51)
Female	172,026 (49)
**Race/ethnicity**	
Asian or Pacific Islander	58,295 (17)
Hispanic	134,453 (38)
Non-Hispanic black	99,363 (28)
Non-Hispanic white	55,857 (16)
**Language spoken at home**	
English	197,727 (57)
Spanish	86,052 (25)
Other language	65,602 (19)
**Place of birth**	
United States	289,160 (83)
Foreign country	60,149 (17)
**Change in fitness^c^ (all years)**	
>20% Decrease	126,115 (19)
10%–20% Decrease	79,172 (12)
<10% Change	253,161 (37)
10%–20% Increase	82,117 (12)
>20% Increase	134,753 (20)
**Change in obesity status^d^ (all years)**	
Obese to not obese	36,029 (5)
Consistently not obese	504,762 (73)
Consistently obese	119,235 (17)
Not obese to obese	27,273 (4)
**School-area poverty^e^**	
Low poverty	62,238 (18)
Medium poverty	119,219 (34)
High poverty	89,407 (26)
Very high poverty	78,510 (22)
**School size**	
Attending small schools (<400 students)	59,856 (17)
Attending nonsmall schools (≥400 students)	289,525 (83)

a N for missing place of birth = 72; N for missing area poverty = 7; N for missing or having >1 race/ethnicity = 177.

b Students in 6th, 7th, and 8th grades contributed 177,281, 220,769, and 186,135 student-years, respectively, across 624 schools.

c Based on change in change in fitness composite percentile scores based on Progressive Aerobic Cardiovascular Endurance Run (PACER) Push-up and Curl-up Fitnessgram tests from the year prior.

d Obesity status was defined according to Centers for Disease Control and Prevention growth chart–derived norms for sex and age (in months), based on a historical reference population, and used to compute the body mass index (BMI) percentile for each child. Obesity was defined as having a BMI ≥95th percentile for youths in the same sex and age (in months) group.

e Based on percentage of households in the school zip code living below the federal poverty threshold (low [<10%], medium [10%–20%], high [>20%–30%], and very high [>30%] area poverty) drawing from the American Community Survey 2007–2012 (27).

**Table 2:** Mean Days Absent per Year Across Student- and School-Level Demographic and Fitness-Change Characteristics in New York City Public Middle School Students (N = 349,381)^a^, 2006–2007 Through 2012–2013

Characteristic	Student-Level^b^, Mean (SD)	School-Level^c^, Mean (SD)
**Sex**		
Male	11.0 (11.7)	11.2 (11.5)
Female	10.1 (11.0)	10.4 (10.8)
**Race/ethnicity**		
Asian or Pacific Islander	5.5 (7.7)	6.4 (8.3)
Hispanic	12.6 (12.9)	13.3 (13.2)
Non-Hispanic black	12.3 (13.1)	12.8 (13.3)
Non-Hispanic white	10.0 (9.7)	10.7 (10.2)
**Language spoken at home**		
English	11.9 (12.1)	12.0 (11.9)
Spanish	10.9 (11.1)	11.0 (10.9)
Other language	6.0 (7.4)	6.5 (7.5)
**Place of birth**		
United States	11.3 (12.1)	11.7 (11.5)
Foreign country	11.1 (13.8)	8.1 (8.8)
**Change in fitness (all years)^d^**		
>20% Increase	10.3 (11.2)	11.0 (11.6)
10%–20% Increase	10.3 (11.3)	10.8 (11.5)
<10% Change	10.7 (11.9)	11.8 (12.6)
10%–20% Decrease	11.1 (12.2)	11.6 (12.4)
>20% Decrease	11.9 (12.8)	12.7 (13.2)
**Grade^e^**		
Grade 6	10.2 (11.0)	10.8 (11.1)
Grade 7	10.9 (12.5)	11.2 (12.2)
Grade 8	13.1 (14.5)	13.1 (13.6)
**School-area poverty^f^**		
Low poverty	8.5 (9.2)	8.9 (9.3)
Medium poverty	9.5 (10.3)	9.8 (10.2)
High poverty	11.1 (11.7)	11.4 (11.6)
Very high poverty	13.1 (13.3)	13.1 (12.9)
**School size**		
Small schools (<400 students)	12.0 (12.3)	11.8 (11.9)
Non-small schools (≥400 students)	10.3 (11.1)	11.8 (11.0)

a N for missing place of birth = 72; N for missing area poverty = 7; N for missing or having >1 race/ethnicity = 177.

b Student-level columns do not account for school clustering.

c School-level columns account for school clustering.

d Based on change in change in fitness composite percentile scores based on Progressive Aerobic Cardiovascular Endurance Run (PACER) Push-up and Curl-up Fitnessgram tests from the year prior.

e Students in 6th, 7th, and 8th grades contributed 177,281, 220,769, and 186,135 student-years, respectively.

f Based on percentage of households in the school zip code living below the federal poverty threshold (low [<10%], medium [10%–20%], high [>20%–30%], and very high [>30%] area poverty) drawing from the American Community Survey 2007–2012 (27).

**Figure 2:**
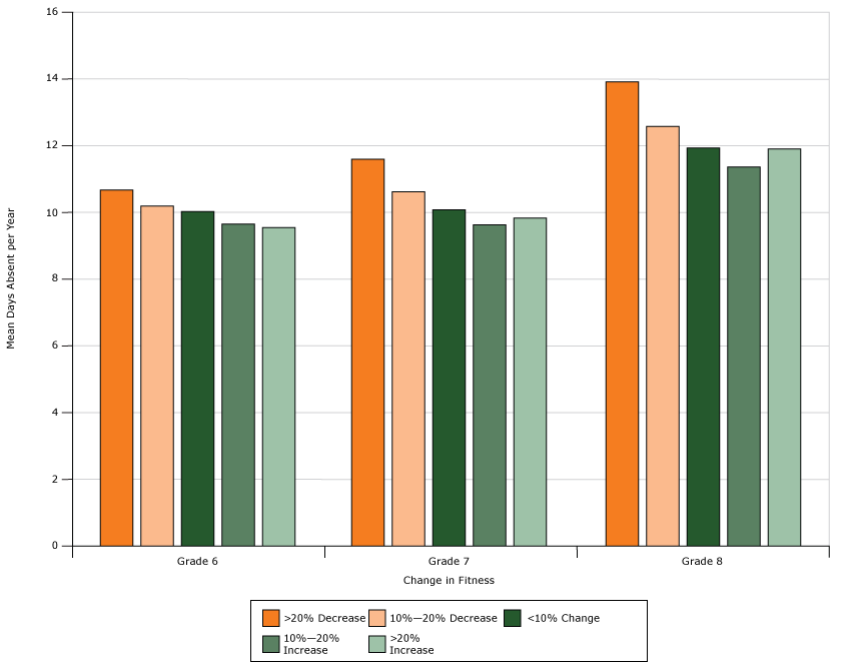
Mean days absent per year by grade across fitness-change categories in New York City public middle school students (N = 349,381), 2006–2007 through 2012–2013. Change in fitness composite percentile scores based on Progressive Aerobic Cardiovascular Endurance Run (PACER) Push-up and Curl-up Fitnessgram tests from the year prior. Categories are based on tabulated mean estimates. **Table:** 

Overall, the mean number of days absent per year decreased with improvements in fitness scores from the year prior. The mean (SD) days absent per year for students with the lowest (>20% decrease) to highest (>20% increase) improvements in fitness were 11.9 (12.8), 11.1 (12.2), 10.7 (11.9), 10.3 (11.3), and 10.3 (11.2). Also, fitness decreased and absenteeism increased with increasing grade (Table 2). Moreover, for students in the same grade, the difference in mean days absent for those with improved versus diminished fitness became larger with increasing grade level (Figure 2). For example, mean (SD) days absent for students with the greatest increase (>20%) in fitness were 9.6 (10.1), 9.8 (10.8), and 11.9 (12.7), for students in 6th, 7th, and 8th grades, respectively. In contrast, mean (SD) days absent for students with the greatest decrease (>20%) in fitness were 10.6 (11.3), 11.6 (12.6), and 13.9 (14.3), for students in 6th, 7th, and 8th grades, respectively.

Fitness Change	Mean Days Absent Per Year		
	Grade 6	Grade 7	Grade 8
>20% Decrease	10.6	11.6	13.9
10%–20% Decrease	10.2	10.6	12.6
<10% Change	10.0	10.1	11.9
10%–20% Increase	9.7	9.7	11.5
>20% Increase	9.6	9.8	11.9

The ICC (model 1) demonstrated a sizable degree of variance in student absenteeism explained by schools (9%). Results from model 2 showed all levels of change in fitness were significantly associated with absenteeism (*P* < .001). Compared with the reference category (>20% decrease in fitness), the absenteeism rate decreased 13.3% (95% CI, 8.3–16.6), 8.3% (95% CI, 3.3–12.7), 5.6% (95% CI, 1.9–9.0), and 1.6% (95% CI, −3.0 to 6.2) for those who had a greater than 20% increase, 10% to 20% increase, less than 10% change, and 10% to 20% decrease in fitness composite percentile scores from the year prior, respectively.

After adjusting for covariates (sex, race/ethnicity, change in obesity status from the year prior, place of birth, SES, and school size), and including interactions (grade*race/ethnicity, grade*sex, grade*place of birth, and SES*race/ethnicity), β estimates for the association of fitness change and lagged number of days absent per year diminished but remained significant (*P* < .005). Relative to the reference category (>20% decrease in fitness), the absenteeism rate decreased 11.9% (95% CI, 7.2–16.8), 6.1% (95% CI, 1.0–11.4), 2.6% (95% CI, −1.1 to 6.5), and 0.4% (95% CI, −4.3 to 5.4) for those who had a greater than 20% increase, 10% to 20% increase, less than 10% change, and 10% to 20% decrease in fitness composite percentile scores from the year prior, respectively (model 3, Table 3).

**Table 3:** Association of Fitness Change and Attendance in New York City Public Middle School Students^a^, 2006–2007 Through 2012–2013

Fitness Change^b^	Unadjusted (Model 2)^c^, IRR^d^ (95% CI)	Adjusted (Model 3)^c^^,^ e, IRR^d^ (95% CI)
>20% Increase	1.13 (1.09–1.18)	1.12 (1.07–1.17)
10%–20% Increase	1.08 (1.03–1.14)	1.06 (1.01–1.11)
<10% Change	1.06 (1.02–1.09)	1.03 (0.989–1.07)
10%–20% Decrease	1.02 (0.97–1.06)	1.00 (0.96–1.05)
>20% Decrease	1 [Reference]	

Abbreviations: CI, confidence interval; IRR, incidence rate ratio.

a N = 349,381 students in 6th, 7th, and 8th grades; 675,318 observations across 624 schools.

b Change in fitness composite percentile scores based on Progressive Aerobic Cardiovascular Endurance Run (PACER) Push-up and Curl-up Fitnessgram tests from the year prior.

c Based on 3-level longitudinal negative binomial mixed models.

d All estimates, *P* < .001.

e Adjusted for sex, race/ethnicity, change in obesity status from the year prior, place of birth (United States or foreign country), school size, and school-area poverty, and including interactions grade*race/ethnicity, grade*sex, grade*place of birth, and school-area poverty*race/ethnicity.

Sensitivity analyses were run to determine the effect of days of enrollment exclusions, BMI categorization specification, and total years of consecutive fitness change data on findings. Results showed slightly more conservative estimates for the magnitude of effects, although the inverse dose–response association remained consistent and significant (*P* < .001, *P* = .004, and *P* = .01 for enrollment, BMI, and fitness data sensitivity models, respectively).

## Discussion

We found that all levels of 1-year change in fitness were significantly associated with absenteeism (*P* < .001) in both crude and adjusted models. Furthermore, consistent levels of fitness improvement each year at the greater than 20% level (vs >20% decrease) were found to have the potential to reduce a student’s number of days absent substantially. For example, a child with a mean 10 days absent in 6th grade would have 6.5 days absent per year in 8th grade and 1.5 days absent per year in 12th grade. This change in days absent represents a shift well within the range of regular attendance (≤5 days absent per year). Findings here are consistent with the existing cross-sectional literature on fitness and absenteeism (4,5,17), lending strong support for future research on the effects of youth fitness interventions on school absenteeism. NYC programs unrelated to fitness promotion have shown a 15% reduction in chronic absenteeism in 100 high-need schools over 2 years (13), through implementing “early warning” flags to identify at-risk students, family and student “success mentors,” progress monitoring systems, and community collaborations. However, despite gains and similar programs nationally, high absenteeism rates remain widespread, including 5 million to 7.5 million chronically absent US students each year (13,14).

Strengths of this study were being the first article to the authors’ knowledge to examine the association of change in fitness and lagged absenteeism, drawing from multiple years of multilevel data. Also, this analysis included a large and diverse study sample of approximately 349,000 students comprised of 6 cohorts.

Findings from this study may not be generalized to other cities or nationally, given a high minority and low-income population in NYC. Future work should examine potential differences in the fitness–attendance relationship by race/ethnicity and poverty status, given higher absenteeism observed in this study among both non-Hispanic black and Hispanic students and those attending schools in high poverty areas. Furthermore, although DOE protocols promote retesting students who are absent on the original testing dates, a large number of students were excluded because of missing Fitnessgram tests for 2 or more consecutive years, insufficient enrollment period, or moving schools. Not all students are required to take the Fitnessgram, including those with chronic health conditions such as severe asthma. These students, however, would be more likely to have higher absenteeism given psychosocial, family, and health factors associated with moving and long-term absences (31). These effects potentially would move the association farther from the null.

Although we offer evidence in support of a causal association between fitness change and absenteeism, a bidirectional relationship may exist between exposure and outcome. For example, it is possible that children who have higher absenteeism are more sedentary, particularly if they are ill or occupied in nonactive ways (eg, video-game playing, watching television). Domestic factors may also persist over time. In this sense, although this analysis lagged absenteeism to fitness, the temporality of exposure and outcome could be reversed. Future research should explore the directionality of fitness and absenteeism in more detail, in addition to the role of chronic conditions in this association.

In our study, systematic bias and differential measurement error are possible, given that the Fitnessgram data are not collected for research purposes. Data were not available on many student- and school-level factors, including self-esteem, drug and alcohol use, family structure, and individual household poverty (such as income or eligibility for free or reduced-price lunch). These factors may influence not only absenteeism but also motivation to perform well on fitness tests. Absence of this data makes it difficult to disentangle these relationships. Future work should research whether mental, social, or emotional health and peer or parent influence are antecedents to fitness on the hypothesized fitness–attendance causal pathway. This research may shed light on why some adolescents have fitness performance drop-offs and may garner particular attendance benefits from these interventions.

Although testing protocols are designed to promote consistency across administers, Fitnessgram testing sites may vary in their implementation of the protocol. However, in NYC the Fitnessgram is administered by physical education teachers who receive formal training on conducting the test, including manuals, video-based training, and site visits, as well as calibrated scales (22,23).

Fitness levels in US youths decline with increasing age at rates faster than in other nations. Diminished fitness is shown in longitudinal studies to be associated with lower academic performance, and cross-sectionally to be associated with higher absenteeism. We present evidence for a longitudinal inverse dose–response association between fitness and absenteeism in NYC middle school youths. Cumulative effects of consistent fitness improvements from 6th through 12th grades may shift a child from chronic absenteeism to regular attendance. Future research should examine the effectiveness of school-based fitness interventions to reduce absenteeism rates, particularly within subgroups that have fitness drop-offs in adolescence. Findings may inform policy mandating increases in school fitness time, including increased classroom-based physical activity and both stricter school physical education and recess policies.
